# Meta-analysis of test accuracy studies: an exploratory method for investigating the impact of missing thresholds

**DOI:** 10.1186/2046-4053-4-12

**Published:** 2015-02-04

**Authors:** Richard D Riley, Ikhlaaq Ahmed, Joie Ensor, Yemisi Takwoingi, Amanda Kirkham, R Katie Morris, J Pieter Noordzij, Jonathan J Deeks

**Affiliations:** Research Institute of Primary Care and Health Sciences, Keele University, Staffordshire, ST5 5BG UK; School of Health and Population Sciences, University of Birmingham, Public Health Building, Edgbaston, Birmingham, B15 2TT UK; MRC Hub for Trials Methodology Research, School of Health and Population Sciences, University of Birmingham, Public Health Building, Edgbaston, Birmingham, B15 2TT UK; Research Section of Reproduction, Genes and Development; School of Clinical and Experimental Medicine, College of Medical and Dental Sciences, University of Birmingham, Birmingham, UK; Fetal Medicine Centre, Birmingham Women’s Hospital NHS Foundation Trust, Birmingham, UK; Department of Otolaryngology - Head & Neck Surgery, Boston Medical Center, Boston University - School of Medicine, Boston, MA USA

**Keywords:** Meta-analysis, Diagnostic test, Multiple thresholds, Imputation, Missing data, Sensitivity analysis

## Abstract

**Background:**

Primary studies examining the accuracy of a continuous test evaluate its sensitivity and specificity at one or more thresholds. Meta-analysts then usually perform a separate meta-analysis for each threshold. However, the number of studies available for each threshold is often very different, as primary studies are inconsistent in the thresholds reported. Furthermore, of concern is selective reporting bias, because primary studies may be less likely to report a threshold when it gives low sensitivity and/or specificity estimates. This may lead to biased meta-analysis results. We developed an exploratory method to examine the potential impact of missing thresholds on conclusions from a test accuracy meta-analysis.

**Methods:**

Our method identifies studies that contain missing thresholds bounded between a pair of higher and lower thresholds for which results are available. The bounded missing threshold results (two-by-two tables) are then imputed, by assuming a linear relationship between threshold value and each of logit-sensitivity and logit-specificity. The imputed results are then added to the meta-analysis, to ascertain if original conclusions are robust. The method is evaluated through simulation, and application made to 13 studies evaluating protein:creatinine ratio (PCR) for detecting proteinuria in pregnancy with 23 different thresholds, ranging from one to seven per study.

**Results:**

The simulation shows the imputation method leads to meta-analysis estimates with smaller mean-square error. In the PCR application, it provides 50 additional results for meta-analysis and their inclusion produces lower test accuracy results than originally identified. For example, at a PCR threshold of 0.16, the summary specificity is 0.80 when using the original data, but 0.66 when also including the imputed data. At a PCR threshold of 0.25, the summary sensitivity is reduced from 0.95 to 0.85 when additionally including the imputed data.

**Conclusions:**

The imputation method is a practical tool for researchers (often non-statisticians) to explore the potential impact of missing threshold results on their meta-analysis conclusions. Software is available to implement the method. In the PCR example, it revealed threshold results are vulnerable to the missing data, and so stimulates the need for advanced statistical models or, preferably, individual patient data from primary studies.

**Electronic supplementary material:**

The online version of this article (doi:10.1186/2046-4053-4-12) contains supplementary material, which is available to authorized users.

## Background

Medical tests are used to inform screening, diagnosis and prognosis in medicine. Meta-analysis methods are increasingly used to synthesise the evidence about a test’s accuracy from multiple studies, to produce summary estimates of sensitivity and specificity [1–4]. When the test is measured on a continuous scale, many studies report test performance at multiple thresholds, each relating to a different choice of threshold above which test results are classed as ‘positive’ and below which test results are classed as ‘negative’. Unfortunately, most primary studies do not report the same set of thresholds. For example, in an evaluation of the spot protein:creatinine ratio (PCR) for detecting significant proteinuria in pregnancy, Morris et al. [5] extracted tables for 23 different thresholds across 13 studies; eight of the thresholds were considered by just one study, but the other 15 thresholds were considered in two or more studies (Table 1), with a maximum of six studies for any threshold. In this situation, meta-analysts generally either utilise the results for just one of the thresholds per study or utilise results for all reported thresholds but perform a separate meta-analysis for each of the thresholds independently [6]. However, an approach that considers meta-analysis for each threshold independently will omit any studies that do not report the threshold of interest and thus also ignore information from other thresholds that are available in those studies.

**Table 1:** **PCR results for each threshold in each of the 13 studies of Morris et al.** [5]

First author	Threshold ID, ***t***	Threshold value, ***x***	TP	FP	FN	TN	Total	High proteinuria	Normal proteinuria
Al Ragib	1	0.13	35	51	4	95	185	39	146
	6	0.18	33	42	6	104			
	7	0.19	33	39	6	107			
	8	0.2	31	38	8	108			
	22	0.49	29	23	10	123			
Durnwald	3	0.15	156	35	12	17	220	168	52
	8	0.2	152	27	16	25			
	15	0.3	136	23	32	29			
	19	0.39	123	14	45	38			
	20	0.4	120	12	48	40			
	23	0.5	106	9	62	43			
Dwyer	3	0.15	54	28	2	32	116	56	60
	5	0.17	51	25	5	35			
	7	0.19	50	18	6	42			
	12	0.24	41	8	15	52			
	14	0.28	37	3	19	57			
	19	0.39	31	0	25	60			
Leonas	15	0.3	277	7	5	638	927	282	645
Ramos	23	0.5	25	1	1	20	47	26	21
Robert	15	0.3	27	4	2	38	71	29	42
Rodriguez	2	0.14	69	34	0	35	138	69	69
	3	0.15	68	34	1	35			
	4	0.16	68	26	1	43			
	5	0.17	65	25	4	44			
	6	0.18	62	24	7	45			
	7	0.19	62	21	7	48			
	8	0.2	60	19	9	50			
	9	0.21	60	17	9	52			
Saudan	8	0.2	14	27	0	59	100	14	86
	13	0.25	13	14	1	72			
	15	0.3	13	7	1	79			
	18	0.35	12	4	2	82			
	20	0.4	11	3	3	83			
	21	0.45	10	0	4	86			
Schubert	3	0.15	9	3	0	3	15	9	6
	4	0.16	9	2	0	4			
Shahbazian	8	0.2	35	2	3	41	81	38	43
Taherian	2	0.14	67	7	6	20	100	73	27
	3	0.15	67	3	6	24			
	4	0.16	65	1	8	26			
	5	0.17	64	1	9	26			
	6	0.18	63	0	10	27			
	8	0.2	59	0	14	27			
Wheeler	9	0.21	59	13	9	45	126	68	58
Yamasmit	7	0.19	29	6	0	7	42	29	13
	9	0.21	29	5	0	8			
	10	0.22	29	4	0	9			
	11	0.23	28	3	1	10			
	12	0.24	28	2	1	11			
	13	0.25	28	1	1	12			
	14	0.28	27	1	2	12			
	16	0.31	26	1	3	12			
	17	0.32	25	1	4	12			

*ID* ordered identification number, *TP* true positives, *FP* false positives, *TN* true negatives, *FN* false negatives.

In this article, we propose an exploratory method (a sensitivity analysis) to help researchers examine the potential impact of missing thresholds on their meta-analysis conclusions about a test’s accuracy. The method first imputes results in studies where missing thresholds are bounded between a pair of known thresholds; missing results are also bounded because as the threshold value increases, sensitivity must decrease and specificity must increase. The imputed results are then added to the meta-analysis, and this allows researchers to evaluate whether their original conclusions are robust. This is especially important when thresholds are prone to selective reporting bias; that is, they are less likely to be reported when they give lower values of sensitivity and/or specificity. In this situation, meta-analysis may otherwise produce summary sensitivity and specificity results that are too high (i.e. biased).

The article is structured as follows. In the “Motivating example” section, we describe the motivating PCR dataset in detail. In the “Methods” section, we describe our imputation method, explain its assumptions and perform an empirical evaluation. The “Results” section applies it to the PCR data, and the “Discussion” section concludes by considering the strengths and limitations of the method and further research.

### Motivating example: identification of significant proteinuria in patients with suspected pre-eclampsia

Pre-eclampsia is a major cause of maternal and perinatal morbidity and mortality and occurs in 2%–8% of all pregnancies [7–10]. The diagnosis of pre-eclampsia is determined by the presence of elevated blood pressure combined with significant proteinuria (≥0.3 g per 24 h) after the 20th week of gestation in a previously normotensive, non-proteinuric patient [11]. The gold-standard method for detection of significant proteinuria is the 24-h urine collection, but this is cumbersome, time consuming and inconvenient, to patients as well as hospital staff. There is therefore a need for a rapid and accurate diagnostic test to identify significant proteinuria to allow more timely decision-making.

The spot PCR has been shown to be strongly correlated with 24-h protein excretion and thus is a potential diagnostic test for significant proteinuria. Morris et al. [5] performed a systematic review and meta-analysis to assess the diagnostic accuracy of PCR for the detection of significant proteinuria in patients with suspected pre-eclampsia. Thirteen relevant studies were identified, and in each study, the reference standard was proteinuria greater than or equal to 300 mg in urine over 24 h. Across the 13 studies, 23 different threshold values were considered for PCR, ranging from 0.13 to 0.50. Five studies provided diagnostic accuracy results (i.e. a two-by-two table showing the number of true positives, false positives, false negatives and true negatives) for just one threshold, but the other eight studies reported results for each of multiple thresholds, up to a maximum of nine thresholds (Yamasmit study). Eight of the 23 thresholds were considered by just one study, but the other 15 thresholds were considered in two or more studies, up to a maximum of six studies (for threshold 0.20). The studies and thresholds are summarised in Table 1.

Meta-analysis is important here to summarise the diagnostic accuracy of PCR at each threshold from all the published evidence, to help ascertain whether PCR is a useful diagnostic test and, if so, which threshold is the most appropriate to use in clinical practice. However, this is non-trivial given the number of thresholds available, the variation in how many studies report each threshold and the likely similarity between neighbouring threshold results. The PCR data is thus an ideal dataset to motivate and apply the statistical methods developed during the remainder of the paper.

## Methods

We now propose our exploratory method for examining the impact of missing thresholds in meta-analysis of test accuracy studies.

### Exploratory method to examine the potential impact of missing thresholds

Let there be *i* = 1 to *m* studies that measure a continuous test result on *n*_1*i*_ diseased patients and *n*_0*i*_ non-diseased patients, whose true disease status is provided by a reference standard. In each study, at a particular threshold value, *x*, each patient’s measured test value is classed as either ‘positive’ (≥ *x*) or ‘negative’ (<*x*). Then summarising test results over all patients produces aggregate data in the form of *r*_11*ix*_, the number of truly diseased patients in study *i* with a positive test result at threshold *x*, and *r*_00*ix*_, the number of non-diseased patients in study *i* with a negative test result. The observed sensitivity at threshold *x* in each study is thus simply *r*_11*ix*_/*n*_1*i*_ and the observed specificity is *r*_00*ix*_/*n*_0*i*_.

When results for a particular threshold are missing but other thresholds above and below are available, then the missing threshold has sensitivity and specificity results constrained between these values. For example, consider the Al Ragib study (Table 1), which has threshold values of 0.13 and 0.18 available, but not 0.14 to 0.17. The number of true positives for the missing thresholds must be constrained between the other threshold values of 35 and 33. Similarly, the missing false positives must be within 42 and 51, the missing false negatives within 4 and 6 and the missing true negatives within 95 and 104.

Rather than ignoring missing thresholds that are bounded between known thresholds, our exploratory method imputes the missing results under particular assumptions, so that they can be included in the meta-analysis. The aim is to ascertain whether the original meta-analysis conclusions (obtained without imputed data) are robust to the inclusion of imputed data. For example, does the summary test accuracy at each threshold remain similar, and is the choice of best threshold the same? The exploratory method is a two-step approach, as now described.

### Step 1: imputation of missing bounded thresholds in each study

**Figure 1:**
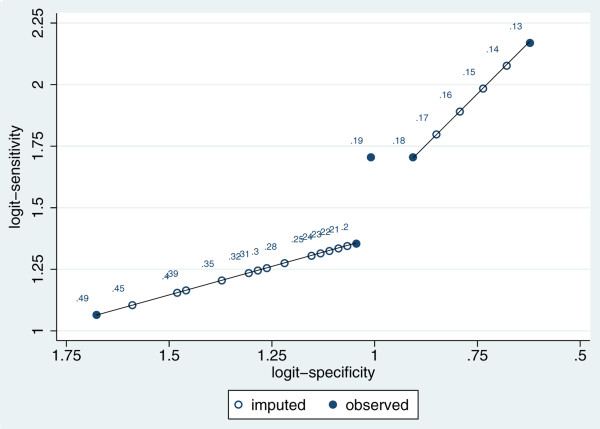
**Graphical illustration of the imputation approach for the Al Ragib study.**

In each study separately, for each threshold that is missing but bounded between known thresholds, the missing results are imputed by assuming each 1-unit increase in threshold value corresponds to a constant reduction in logit-sensitivity (*y*_1*ix*_), and also a constant increase in logit-specificity (*y*_0*ix*_). Thus, imputation is on a straight line between pairs of observed points in logit receiver operating characteristic (ROC) space. This piece-wise linear approach is illustrated graphically in Figure 1. So the key assumption here is a constant change in logit values for each 1-unit change in threshold value between each pair of observed threshold results. The linear slope can be different between each pair of thresholds, and so no single trend is assumed across all thresholds, with the fitted lines forced to go through the observed points. Linear relationships on the logit scale are often used in diagnostic test analyses when considering the ROC curve, especially in meta-analysis [12], and it is a straightforward approach for this exploratory analysis.

Once the imputed logit values are obtained, one can back transform to compute the corresponding imputed true and false positives and negatives. For example, let TP1 be the true positive number at a threshold value of 0.13 and TP2 be the true positive number at threshold 0.18. Then, in the Al Ragib study there are 5 threshold units from 0.13 to 0.18. The imputed logit-sensitivity at threshold 0.14 is *y*_1*i*(0.13)_ + (*y*_1*i*(0.18)_ - *y*_1*i*(0.13)_)/5, and at threshold 0.15 the imputed logit-sensitivity is *y*_1*i*(0.13)_ + (2(*y*_1*i*(0.18)_ - *y*_1*i*(0.13)_)/5), and so on (Table 2, Figure 1). It is then straightforward to calculate the number of true positives, false positives, true negatives and false negatives that are necessary to produce these values. For example, for threshold 0.15, the imputed logit-sensitivity is 1.983, and so the imputed sensitivity is 0.879, and given there are 39 patients truly with high proteinuria, the imputed number of true positives and false negatives is 34.28 and 4.72, respectively (Table 2).

**Table 2:** **Actual and imputed results for the Al Ragib study between thresholds 0.13 and 0.18**

First author	Threshold ID, ***t***	Threshold value, ***x***	Imputed?	TP	FP	FN	TN	Total	High proteinuria	Normal proteinuria
Al Ragib	1	0.13	No	35	51	4	95	185	39	146
	2	0.14	Yes	34.7	49.1	4.3	96.9			
	3	0.15	Yes	34.3	47.3	4.7	98.7			
	4	0.16	Yes	33.9	45.5	5.1	100.5			
	5	0.17	Yes	33.5	43.7	5.5	102.3			
	6	0.18	No	33	42	6	104			

The imputation is undertaken on the logit-scale, and then the values are back transformed to calculate the corresponding imputed raw data values.

*ID* ordered identification number, *TP* true positives, *FP* false positives, *TN* true negatives, *FN* false negatives.

Note that we neither impute beyond the highest available threshold nor impute below the lowest available threshold in each study. Further assumptions would be necessary to do this, but here we only work within the limits of the observed data available. Thus, for studies with only 1 threshold reported, no imputation was used. Similarly, we do not impute for any new threshold values which were not considered by any of the available studies.

A STATA ‘do’ file to fit the imputation method is available in Additional file 1, and we aim to release an associated STATA module in the near future. It provides the original and imputed values within a few seconds, for any number of studies and any number of thresholds.

### Step 2: meta-analysis at each threshold separately using actual and imputed data

The imputation in step 1 borrows strength from available thresholds to allow a larger set of threshold data to be available from each study for meta-analysis. For ease of language, let us order the thresholds of interest and refer to the ordered value as *t* (e.g. *t* = 1 to 23 in the PCR example, Table 1)*.* Each threshold *t* now has (i) one or more studies with observed results and potentially (ii) some studies with imputed results. A separate meta-analysis of each threshold separately can now be considered, using the observed and imputed results. A convenient model is the bivariate meta-analysis of Chu and Cole [2]. This approach is recommended by the Cochrane Screening and Diagnostic Test Methods Group and is commonly used in diagnostic accuracy meta-analyses. It utilises the exact binomial within-study distribution, thereby avoiding the need for any continuity corrections, and accounts for any between-study correlation in sensitivity and specificity, as follows:
1

*β*_1*t*_ and *β*_0*t*_ give the average logit-sensitivity and average logit-specificity at threshold *t*, respectively, and these can be transformed to give the summary sensitivity and summary specificity from the meta-analysis for each threshold. The between-study covariance matrix is given by **Ω**_*t*_, containing the between-study variances ( and ) and the between-study correlation in logit-sensitivity and logit-specificity (*ρ*_10*t*_); if the latter is zero, the model reduces to a separate univariate analysis for each of sensitivity and specificity. Indeed, *ρ*_10*t*_ will often be poorly estimated at +1 or -1 [13], and so it may be sensible to adopt two separate univariate models here [14], as follows:
2

Models (1) and (2) can be estimated using adaptive Gaussian quadrature [15], for example using PROC NLMIXED in SAS [16], or the xtmelogit command in STATA [17]. A number of quadrature points can be specified, with increasing estimation accuracy as the number of points increases, but at the expense of increased computational time. We generally chose 5 quadrature points for our analyses, as this gave estimates very close to those when using >10 points but in a faster time. Successful convergence of the optimization procedure was assumed when successive iteration estimates differed by <10^-7^, resulting in parameter estimates and their approximate standard errors based on the second derivative matrix of the likelihood function.

### Empirical evaluation of the imputation method

To empirically evaluate the imputation method, we utilised individual participant data (IPD) from six studies examining the ability of parathyroid hormone (PTH) to correctly classify which patients will become hypocalcemic within 24-h after a thyroidectomy [18]. The percentage decrease in PTH (from pre-surgery to 6-h post-surgery) was used as the test, and thresholds of 40%, 50%, 60%, 65%, 70%, 80% and 90% were examined. As IPD were available, the results for all thresholds were available for all studies. Thus, for each threshold separately, we could fit model (1) using the complete set of data from the six studies. However, model (1) often poorly estimated the between-study correlations at +1 or -1 and gave summary test accuracy results very similar to model (2). Thus we focus here only on model (2) results, and these provided our ‘complete data’ meta-analysis results, for when the thresholds are all truly available from all studies.

#### Generation of missing thresholds and imputation

To replicate missing data mechanisms, we considered two scenarios:

Scenario (i): thresholds missing at random We took the complete set of threshold results for each study and randomly assigned some to be missing, with each having a 0.5 probability of being omitted. This provided a new meta-analysis dataset of up to six studies with missing threshold results. We repeated this process until 1,000 such meta-analysis datasets had been produced. In each dataset, we applied our imputation approach, and then for each threshold, we fitted model (2) to (i) each generated dataset without including the imputed results and (ii) each generated dataset with the addition of the imputed results. We then compared the average meta-analysis estimates and standard errors from the 1,000 analyses of (i) and (ii) with the true meta-analysis results when the complete data were available (Table 3).

**Table 3:** **Empirical evaluation results—scenario (i), thresholds missing at random**

% PTH decrease	Estimate of interest	Meta-analysis of the complete data	Meta-analysis of the datasets with missing threshold results, not including imputed results		Meta-analysis of the datasets with missing threshold results, including imputed results	
		True estimate	Mean estimate across the 1,000 datasets	Median estimate across the 1,000 datasets	Mean estimate across the 1,000 datasets	Median estimate across the 1,000 datasets
40	Summary Sensitivity	0.87	0.88	0.90	0.88	0.90
		0.00	0.04	0.00	0.04	0.00
	s.e. (logit sensitivity)	0.40	0.70	0.62	0.70	0.62
	Summary specificity	0.52	0.52	0.52	0.52	0.52
		0.00	0.05	0.00	0.05	0.00
	s.e. (logit specificity)	0.18	0.25	0.24	0.25	0.24
50	Summary sensitivity	0.87	0.88	0.90	0.87	0.87
		0.00	0.04	0.00	0.04	0.00
	s.e. (logit sensitivity)	0.40	0.69	0.62	0.54	0.47
	Summary specificity	0.62	0.63	0.63	0.64	0.63
		0.00	0.05	0.01	0.04	0.00
	s.e. (logit specificity)	0.18	0.27	0.26	0.22	0.21
60	Summary sensitivity	0.87	0.88	0.90	0.87	0.86
		0.00	0.04	0.00	0.05	0.00
	s.e. (logit sensitivity)	0.40	0.69	0.62	0.52	0.43
	Summary specificity	0.78	0.78	0.79	0.78	0.77
		0.00	0.05	0.00	0.05	0.00
	s.e. (logit specificity)	0.21	0.31	0.29	0.24	0.23
65	Summary sensitivity	0.85	0.87	0.89	0.85	0.85
		0.00	0.04	0.00	0.05	0.00
	s.e. (logit sensitivity)	0.38	0.69	0.62	0.49	0.39
	Summary specificity	0.80	0.81	0.82	0.81	0.80
		0.00	0.05	0.00	0.04	0.00
	s.e. (logit specificity)	0.22	0.33	0.32	0.26	0.23
70	Summary sensitivity	0.85	0.86	0.88	0.83	0.84
		0.00	0.07	0.00	0.05	0.00
	s.e. (logit sensitivity)	0.38	0.68	0.64	0.47	0.39
	Summary specificity	0.85	0.86	0.86	0.85	0.85
		0.00	0.04	0.00	0.04	0.00
	s.e. (logit specificity)	0.25	0.36	0.35	0.30	0.26
80	Summary sensitivity	0.73	0.73	0.76	0.72	0.72
		0.00	0.04	0.00	0.05	0.00
	s.e. (logit sensitivity)	0.30	0.49	0.45	0.38	0.35
	Summary specificity	0.91	0.92	0.92	0.91	0.91
		0.00	0.04	0.00	0.04	0.00
	s.e. (logit specificity)	0.30	0.50	0.46	0.41	0.35
90	Summary sensitivity	0.55	0.51	0.55	0.51	0.55
		0.00	0.05	0.00	0.05	0.00
	s.e. (logit sensitivity)	0.27	0.46	0.36	0.46	0.36
	Summary specificity	0.96	0.96	0.96	0.96	0.96
		0.00	0.04	0.00	0.04	0.00
	s.e. (logit specificity)	0.46	0.69	0.59	0.69	0.59

NB All meta-analyses used model (2), as model (1) often poorly estimated *ρ*_10*t*_ as +1 or -1.

Scenario (ii): thresholds selectively missing We took the complete set of threshold results for each study and assigned some to be missing through a selective (not missing at random) mechanism, based on the observed sensitivity estimate. All thresholds with the observed sensitivity ≥ 90% were always included; however, those with sensitivity <90% had a 0.5 probability of being omitted. This reflects a realistic situation where researchers are more likely to report those thresholds where sensitivity is observed to be high. We repeated this process until 1000 such meta-analysis datasets had been produced. In each dataset, we applied our imputation approach, and then for each threshold, we fitted model (2) to (i) each generated dataset without including the imputed results and (ii) each generated dataset with the addition of the imputed results. We then compared the average meta-analysis estimates and standard errors from the 1,000 analyses of (i) and (ii) with those true meta-analysis results when the complete data were available (Table 4).

**Table 4:** **Empirical evaluation results—scenario (ii), thresholds selectively missing**

% PTH decrease	Estimate of interest	Meta-analysis of the complete data	Meta-analysis of the datasetswith missing threshold results, not including imputed results		Meta-analysis of the datasets with missing threshold results, including imputed results	
		True estimate	Mean estimate across the 1,000 datasets	Median estimate across the 1,000 datasets	Mean estimate across the 1,000 datasets	Median estimate across the 1,000 datasets
40	Summary sensitivity	0.87	0.89	0.88	0.89	0.88
		0.00	0.00	0.00	0.00	0.00
	s.e. (logit sensitivity)	0.40	0.52	0.48	0.52	0.48
	Summary specificity	0.52	0.52	0.52	0.52	0.52
		0.00	0.05	0.04	0.05	0.04
	s.e. (logit specificity)	0.18	0.25	0.24	0.25	0.24
50	Summary sensitivity	0.87	0.89	0.90	0.88	0.87
		0.00	0.00	0.00	0.01	0.00
	s.e. (logit sensitivity)	0.40	0.53	0.52	0.46	0.44
	Summary specificity	0.62	0.64	0.64	0.64	0.64
		0.00	0.04	0.04	0.01	0.00
	s.e. (logit specificity)	0.18	0.26	0.26	0.21	0.20
60	Summary sensitivity	0.87	0.89	0.90	0.87	0.87
		0.00	0.00	0.00	0.03	0.00
	s.e. (logit sensitivity)	0.40	0.52	0.48	0.44	0.40
	Summary specificity	0.78	0.79	0.79	0.77	0.77
		0.00	0.03	0.00	0.01	0.00
	s.e. (logit specificity)	0.21	0.28	0.26	0.23	0.21
65	Summary sensitivity	0.85	0.87	0.86	0.85	0.85
		0.00	0.06	0.00	0.04	0.00
	s.e. (logit sensitivity)	0.38	0.54	0.49	0.41	0.38
	Summary specificity	0.80	0.82	0.82	0.81	0.80
		0.00	0.01	0.00	0.00	0.00
	s.e. (logit specificity)	0.22	0.31	0.28	0.24	0.23
70	Summary sensitivity	0.85	0.87	0.86	0.83	0.84
		0.00	0.06	0.00	0.08	0.00
	s.e. (logit sensitivity)	0.38	0.54	0.49	0.41	0.38
	Summary specificity	0.85	0.86	0.85	0.84	0.84
		0.00	0.00	0.00	0.00	0.00
	s.e. (logit specificity)	0.25	0.33	0.31	0.26	0.25
80	Summary sensitivity	0.73	0.75	0.75	0.71	0.71
		0.00	0.02	0.00	0.05	0.00
	s.e. (logit sensitivity)	0.30	0.45	0.41	0.37	0.34
	Summary specificity	0.91	0.90	0.90	0.91	0.91
		0.00	0.00	0.00	0.02	0.00
	s.e. (logit specificity)	0.30	0.41	0.39	0.36	0.35
90	Summary sensitivity	0.55	0.58	0.56	0.58	0.56
		0.00	0.02	0.00	0.02	0.00
	s.e. (logit sensitivity)	0.27	0.41	0.37	0.41	0.37
	Summary specificity	0.96	0.95	0.96	0.95	0.96
		0.00	0.00	0.00	0.00	0.00
	s.e. (logit specificity)	0.46	0.57	0.59	0.57	0.59

NB All meta-analyses used model (2), as model (1) often poorly estimated *ρ*_10*t*_ at +1 or -1.

## Results

### Results of empirical evaluation

In the empirical evaluation, there is no imputation for the lowest and highest threshold of 40% and 90%, and so meta-analysis results are identical for these thresholds regardless of whether imputed data is included or not. However, for other thresholds, there is always potential for imputation.

For thresholds between 40% and 90% in scenario (i), where thresholds were missing at random, the mean and median estimates tend to be slightly closer to the complete data results when using the imputed data (Table 3). For example, at threshold 65%, the true sensitivity estimate from complete data is 0.85, whilst the mean/median without using imputed data is 0.87/0.89, but when using the imputed data, it is pulled back to 0.85/0.85. Further, the meta-analyses including the imputed data give substantially smaller standard errors than those from the meta-analyses excluding imputed data. For example, for threshold 65%, the standard error of the summary logit-sensitivity is 0.69 when ignoring imputed data and 0.49 when including it, a gain in precision of almost 30%. The gain in standard error reflects the additional information being used from the imputed results. As estimates are close to the true estimates and standard errors are considerably reduced, the mean-square error of estimates is therefore improved. The imputation approach also gives standard errors of estimates that are also closer (but not smaller) to those from the true complete data meta-analysis.

For scenario (ii), where thresholds are selectively missing based on the value of observed sensitivity, the summary meta-analysis results without the imputed data are again slightly larger than the true estimated values, especially for sensitivity. The meta-analysis results using the imputed data generally reduce this bias and give more conservative estimates. For example, for the 50% threshold, the true estimated value for sensitivity is 0.87, whilst the median meta-analysis estimate without imputation data is 0.90, but the median estimate including imputed data is pulled back down to 0.87. Occasionally, the imputation method over-adjusted, so that the estimate was pulled down too far. For example, for the 80% threshold, the true estimated value for sensitivity is 0.73, but the median estimate from the without imputation data is 0.75 and the median estimate from the imputed data is 0.71. However, even here the absolute magnitude of bias is the same (0.02) with and without imputed data. For all thresholds between 40% and 90%, there is again considerable reduction in the standard error of meta-analysis estimates following the use of imputed data.

In summary, the empirical evaluation shows that the imputation method performs well, with summary test accuracy estimates generally moved slightly closer to the true estimates based on complete data. This finding, combined with smaller standard errors and thus smaller mean-square error of estimates, suggests the imputation approach is useful as an exploratory analysis.

### Application to the PCR example

Our imputation approach was applied to the PCR studies introduced in the “Motivating example” section, and missing threshold results could be imputed in 6 of the 13 studies. For 21 of the 23 different thresholds, the imputation approach increased the number of studies providing data for that threshold (Table 5), and in total, an additional 50 thresholds results were imputed, substantially increasing the information available for meta-analysis. For example, at a PCR threshold of 0.22, the imputation increased the available studies from 1 to 5, and at a threshold of 0.3, the available studies increased from 4 to 7.

**Table 5:** **Summary meta-analysis results following application of model (2) with and without the imputed data included**

Without imputed data						With imputed data				
Threshold value, ***x***	No. studies with this threshold	Summary estimate	95% CI		Tau	No. studies with this threshold	Summary estimate	95% CI		Tau
								Lower	Upper	
			Lower	Upper						
Sensitivity										
0.13	1	0.897	0.756	0.961	0.000	1	0.897	0.756	0.961	0.000
0.14	2	0.910	0.841	0.951	0.000	3	0.955	0.801	0.991	1.147
0.15	5	0.944	0.901	0.969	0.067	6	0.937	0.909	0.957	0.001
0.16	3	0.960	0.831	0.991	0.850	6	0.925	0.890	0.950	0.091
0.17	3	0.909	0.860	0.942	0.000	5	0.911	0.879	0.935	0.000
0.18	3	0.873	0.816	0.914	0.000	5	0.894	0.860	0.920	0.000
0.19	4	0.902	0.851	0.936	0.000	6	0.889	0.855	0.917	0.096
0.2	6	0.875	0.828	0.910	0.234	8	0.886	0.839	0.921	0.312
0.21	3	0.892	0.834	0.931	0.000	7	0.882	0.848	0.909	0.000
0.22	1	0.983	0.782	0.999	0.000	5	0.899	0.761	0.961	0.669
0.23	1	0.950	0.786	0.990	0.000	5	0.870	0.766	0.932	0.534
0.24	2	0.877	0.575	0.974	0.973	5	0.850	0.756	0.912	0.473
0.25	2	0.953	0.832	0.988	0.000	5	0.850	0.759	0.910	0.442
0.28	2	0.818	0.531	0.947	0.837	5	0.818	0.715	0.890	0.473
0.3	4	0.938	0.829	0.979	0.975	7	0.893	0.773	0.954	1.076
0.31	1	0.883	0.713	0.959	0.000	5	0.780	0.689	0.851	0.362
0.32	1	0.850	0.675	0.939	0.000	5	0.781	0.687	0.853	0.363
0.35	1	0.833	0.562	0.951	0.000	4	0.733	0.641	0.809	0.296
0.39	2	0.662	0.530	0.772	0.320	4	0.699	0.607	0.778	0.278
0.4	2	0.720	0.650	0.780	0.000	3	0.724	0.661	0.779	0.000
0.45	1	0.700	0.436	0.876	0.000	3	0.691	0.627	0.748	0.000
0.49	1	0.842	0.774	0.893	0.000	2	0.657	0.590	0.718	0.000
0.5	2	0.844	0.433	0.975	1.230	2	0.844	0.433	0.975	1.230
Specificity										
0.13	1	0.651	0.570	0.724	0.000	1	0.651	0.570	0.724	0.000
0.14	2	0.671	0.597	0.736	0.000	3	0.624	0.524	0.714	0.250
0.15	5	0.562	0.366	0.740	0.795	6	0.583	0.421	0.728	0.717
0.16	3	0.803	0.499	0.943	1.026	6	0.661	0.462	0.816	0.910
0.17	3	0.765	0.463	0.925	1.042	5	0.677	0.465	0.834	0.922
0.18	3	0.856	0.436	0.979	1.503	5	0.726	0.458	0.893	1.188
0.19	4	0.708	0.653	0.758	0.000	6	0.720	0.522	0.858	0.961
0.2	6	0.818	0.597	0.931	1.245	8	0.775	0.609	0.884	1.031
0.21	3	0.750	0.672	0.815	0.000	7	0.707	0.635	0.771	0.332
0.22	1	0.692	0.409	0.880	0.000	5	0.705	0.599	0.793	0.438
0.23	1	0.769	0.478	0.924	0.000	5	0.738	0.623	0.828	0.508
0.24	2	0.863	0.764	0.925	0.000	5	0.762	0.636	0.855	0.592
0.25	2	0.848	0.764	0.907	0.000	5	0.798	0.655	0.892	0.724
0.28	2	0.945	0.863	0.979	0.000	5	0.845	0.681	0.933	0.947
0.3	4	0.917	0.703	0.981	1.515	7	0.916	0.793	0.969	1.311
0.31	1	0.923	0.609	0.989	0.000	5	0.886	0.707	0.962	1.183
0.32	1	0.923	0.609	0.989	0.000	5	0.892	0.716	0.964	1.194
0.35	1	0.948	0.876	0.979	0.000	4	0.898	0.701	0.971	1.239
0.39	2	0.980	0.092	1.000	3.125	4	0.933	0.713	0.987	1.556
0.4	2	0.903	0.675	0.977	0.982	3	0.872	0.715	0.949	0.796
0.45	1	0.994	0.915	1.000	0.000	3	0.944	0.587	0.995	1.883
0.49	1	0.842	0.774	0.893	0.000	2	0.838	0.779	0.883	0.000
0.5	2	0.863	0.764	0.925	0.000	2	0.863	0.764	0.925	0.000

NB All meta-analyses used model (2), as model (1) often poorly estimated *ρ*_10*t*_ at +1 or -1.

**Figure 2:**
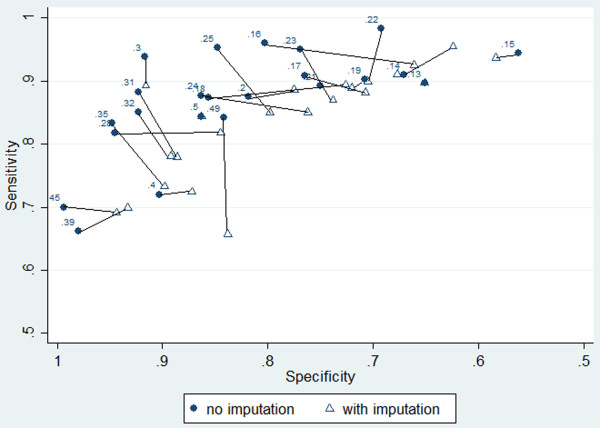
**Summary meta-analysis results presented in ROC space, comparing the summary meta-analysis results shown in Table** 5 **, with and without inclusion of imputed thresholds.** To help compare approaches, summary estimates for the same threshold are shown connected.

Meta-analysis model (1) was applied to each threshold’s data separately, but the between-study correlations were often estimated poorly as -1 in these analyses, so we decided to rather fit model (2) (i.e. *ρ*_10*t*_ was set to zero for all analyses, allowing a separate analysis for sensitivity and specificity at each threshold) [14]. The summary meta-analysis results when including or ignoring the imputed data are shown for each threshold in Table 5 and Figure 2.Importantly, the results when including the imputed data are often very different to when ignoring it. In particular, the summary estimates of sensitivity and specificity are generally reduced when using the imputed data, as can be seen visually in the summary ROC space (Figure 2). For example, when imputed data were included, the summary specificity at a PCR threshold of 0.16 reduced from 0.80 to 0.66 and the summary sensitivity at a PCR threshold of 0.25 reduced from 0.95 to 0.85.

The points in ROC space tend to move down and to the right after including imputed data, revealing lower sensitivity and specificity than previously thought. Thus, it appears that the results when ignoring imputed data may be optimistic, potentially due to biased availability of thresholds when they give higher test accuracy results. In both analyses (assuming sensitivity and specificity are equally important), the best threshold appears to be between 0.25 and 0.30; however, test accuracy at these thresholds is lower after imputation.

The dramatic change in results for some thresholds suggests that individual patient data are needed to obtain a complete set of threshold results from each study and thereby remove the suspected reporting bias in primary studies. We also attempted to use the advanced statistical modelling framework of Hamza et al. [12] to reduce the impact of missing thresholds by jointly synthesising all thresholds in one multivariate model; however, this approach failed to converge, most likely due to the amount of missing data. The multiple thresholds model of Putter et al. [19] also required complete data for all thresholds, whilst the method of Dukic and Gatsonis [20] was not considered suitable, as it produces a summary ROC curve but does not give meta-analysis results for each threshold.

## Discussion

Primary study authors often do not use the same thresholds when evaluating a medical test and will predominately report those thresholds that produce the largest (optimal) sensitivity and specificity estimates [21]. This may lead to optimistic and misleading meta-analysis results based only on reported thresholds. We have proposed an exploratory method for examining the impact of missing threshold results in meta-analysis of test accuracy studies and shown its potential usefulness through an applied example and empirical evaluation. The imputation method is applicable when studies use the same (or similarly validated or standardised) methods of measuring a continuous test (e.g. blood pressure or a continuous biomarker, like prostate-specific antigen). It is deliberately very simple, so that applied researchers can still implement standard meta-analysis methods and examine the potential impact of missing thresholds on meta-analysis conclusions. For example, our application to the PCR data showed how the imputation method revealed lower diagnostic test accuracy results than a standard meta-analysis of each threshold independently, but conclusions about the best choice of threshold appeared robust.

Other more sophisticated methods are also available to deal with multiple thresholds, but all have limitations. Hamza et al. [12] propose a multivariate random-effect meta-analysis approach and apply it when *all* studies report *all* of the thresholds of interest. It models the (linear) relationship between threshold value and test accuracy within each study but is prone to convergence problems (as we experienced for the PCR example), prompting Putter et al. [19] to propose an alternative survival model framework for meta-analysing the multiple thresholds. However, this also requires the multiple thresholds to be available in all studies. Others have also considered the multiple threshold issue [20, 22–27]. A well-known method by Dukic and Gatsonis [20] only produces a summary ROC curve, rather than summary results for each threshold of interest. We recently proposed a multivariate-normal approximation to the Hamza et al. approach [27], which produces both a summary ROC curve and summary results for each threshold and easily accommodates studies with missing thresholds. However, the multivariate-normal approximation to the exact multinomial likelihood is a potential limitation.

Our exploratory method is not a competitor to these more sophisticated methods. Rather, it is an *exploratory* tool aimed at researchers (usually non-statisticians) conducting systematic reviews of test accuracy studies. The method is practical and easy to implement without advanced statistical expertise and so can quickly flag whether researchers should be concerned about missing thresholds in their meta-analysis. This was demonstrated in the PCR example, where the method flagged major concerns that original conclusions were optimistic. In this situation, researchers should be stimulated to put resources toward undertaking the aforementioned advanced statistical methods or, ideally, obtaining individual participant data to calculate missing threshold results directly.

The key reason that we label our method as ‘exploratory’ is that it only considers single imputation. Single imputation of missing values usually causes standard errors of estimates to be too small, since it fails to account for the uncertainty in the imputed values themselves, and multiple imputations would help address this [28]. In particular, imputed data between two thresholds close together (e.g. imputing data for a threshold of 0.24 using available thresholds 0.23 and 0.25) should have less uncertainty than imputing data between two thresholds far apart (e.g. imputing at a threshold of 0.24 using thresholds 0.13 and 0.50), but this is not currently accounted for in our approach. Further research may consider extension to multiple imputations. Also, our imputation assumes a linear relationship between threshold value and logit-sensitivity and logit-specificity; although this linear relationship is commonly used in meta-analysis of test accuracy studies, it is of course an assumption.

Thus, our imputation method is a sensitivity analysis: it shows, under the assumptions made, how vulnerable the original meta-analysis conclusions are to the missing threshold results. The focus is therefore on how the method modifies the original summary meta-analysis estimates; less attention should be paid to the standard errors and confidence intervals it produces, as these may be artificially small and narrow. The method is thus similar in spirit to how others have evaluated the potential impact of (biased) missing data in meta-analysis of randomised trials, such as trim and fill [29] and adjustments based on funnel plot asymmetry [30]. For example, trim and fill imputes missing studies assuming asymmetry is caused by publication bias and Peters et al.[31] conclude it ‘can help to reduce the bias in pooled estimates, even though the performance of this method is not ideal … we recommend use of the trim and fill method as a form of sensitivity analysis.’ Similarly, our method can help to reduce bias and mean-square error in pooled meta-analysis results.

## Conclusion

We have proposed an exploratory analysis that allows researchers to examine the potential impact of missing thresholds on the conclusions of a test accuracy meta-analysis. Currently, most researchers ignore this issue, but our PCR example shows that this may be naive, as conclusions are susceptible to selective threshold reporting in primary studies. STATA code to fit the imputation approach is available in the Additional file 1, and an associated STATA module will be released in the near future.

## Electronic supplementary material

Additional file 1: **A STATA ‘do’ file to fit the proposed imputation method.** (ZIP 6 KB)
